# Dynamics of manual impaction instruments during total hip arthroplasty

**DOI:** 10.1302/2046-3758.134.BJR-2023-0224.R1

**Published:** 2024-04-23

**Authors:** Adam Reynolds, Ruben Doyle, Oliver Boughton, Justin Cobb, Sarah Muirhead-Allwood, Jonathan Jeffers

**Affiliations:** 1 Biomechanical Engineering, Imperial College London, London, UK; 2 Surgery & Cancer, Imperial College London, London, UK; 3 Orthopaedics, Imperial College London, London, UK; 4 The London Hip Unit, Princess Grace Hospital, London, UK; 5 Department of Mechanical Engineering, Imperial College London, London, UK

**Keywords:** Hip replacement, Surgical technique, Motion capture, Mallet, total hip arthroplasty (THA), Acetabular component, consultant orthopaedic surgeon, cementless acetabular cups, acetabulum, hip, Upper limb injuries, surgical approach, hip arthroplasty surgeries, variance

## Abstract

**Aims:**

Manual impaction, with a mallet and introducer, remains the standard method of installing cementless acetabular cups during total hip arthroplasty (THA). This study aims to quantify the accuracy and precision of manual impaction strikes during the seating of an acetabular component. This understanding aims to help improve impaction surgical techniques and inform the development of future technologies.

**Methods:**

Posterior approach THAs were carried out on three cadavers by an expert orthopaedic surgeon. An instrumented mallet and introducer were used to insert cementless acetabular cups. The motion of the mallet, relative to the introducer, was analyzed for a total of 110 strikes split into low-, medium-, and high-effort strikes. Three parameters were extracted from these data: strike vector, strike offset, and mallet face alignment.

**Results:**

The force vector of the mallet strike, relative to the introducer axis, was misaligned by an average of 18.1°, resulting in an average wasted strike energy of 6.1%. Furthermore, the mean strike offset was 19.8 mm from the centre of the introducer axis and the mallet face, relative to the introducer strike face, was misaligned by a mean angle of 15.2° from the introducer strike face.

**Conclusion:**

The direction of the impact vector in manual impaction lacks both accuracy and precision. There is an opportunity to improve this through more advanced impaction instruments or surgical training.

Cite this article: *Bone Joint Res* 2024;13(4):193–200.

## Article focus

- This study examines the relative motion of a surgical mallet, in relation to the introducer, during acetabular component impaction performed by a consultant surgeon on cadaveric samples.
- Three parameters, namely strike vector angle, mallet face angle, and strike offset, were analyzed to assess the precision and accuracy of the strike.

## Key messages

- Mallet strikes were consistently found to be inaccurate and imprecise in all three measured parameters, indicating misalignment and offset from the central axis of the introducer.
- There may be an opportunity to improve these parameters through more advanced impaction instruments or surgical training.

## Strengths and limitations

- To our knowledge, this study is the first of its kind to analyze the accuracy of mallet striking motion instead of focusing solely on delivered force. It brings attention to a previously overlooked aspect of surgical technique that can be enhanced.
- It is important to note that this study was conducted in a controlled laboratory setting using cadaveric samples and a single surgeon. Factors such as inter-surgeon variation and the challenges posed by the surgical environment could not be accounted for.

## Introduction

Over the last 20 years, there has been a trend toward the use of cementless acetabular cups in hip arthroplasties.^1-3^ During 2021 in the UK, three-quarters of primary hip arthroplasty surgeries used either a full cementless system (cementless cup and femoral stem) or a hybrid system (cementless cup i and cemented femoral stem).^1^ The use of cementless cup fixation is even more widespread in the USA and Australia, used in over 90% of cases in 2020.^2,3^ Cementless cups are preferred for their long-term survivorship, which is achieved as a result of bone ingrowth.^1,4^ However, this can only occur if adequate short-term stability has been achieved through the initial press-fit of the cup.^5-9^ This process of impaction is therefore an important skill to optimize.

The standard method of impacting the acetabular component is with a weighted surgical mallet and introducer. Characterization of this method has gained traction over recent years. Previous studies have characterized the forces involved in acetabular component impaction during total hip arthroplasty (THA), with reported average impaction forces ranging between 3.2 kN and 30.3 kN.^10-13^ These differences in reported impaction forces may be due to differences in the set-up such as bone model and load cell position. However, a common theme of all studies is high variability between strikes and high variability between surgeons.

While impaction forces are well understood, the direction of the force vector has yet to be reported. Acetabular component fixation testing is often performed in drop tower test rigs or uniaxial testing rigs where the force application is perfectly linear.^14-20^ However, this is not a perfect model of surgery. The action of swinging a hammer involves coordination of several joints and muscle groups, and may introduce uncontrolled rotational movement of the cup in addition to the desired linear translation. Variability may relate to the surgeon’s dominant hand, whether the hip is left or right, and the surgical approach used. Any off-axis component is undesirable, as uncontrolled movement will serve to damage the bone interface unnecessarily.^21^

The ideal strike vector to seat an acetabular component should be accurate and precise. For accuracy, the strike should be aligned along the axis of the introducer, directing the cup into position without wasting the surgeon’s energy or introducing rotational interfacial micromotion that could reduce the initial stability of the cup. For precision, the variability of the direction of the strike vector should be minimized. This study aims to quantify the accuracy and precision of manual impaction strikes during the seating of an acetabular component. This understanding will help us to improve impaction surgical techniques and inform the development of future technologies.

## Methods

### Data capture

Approval was given by Imperial College London’s Research Ethics Committee to conduct a study on three recently deceased, phenol soft-fixed cadavers (mean age 87 years, mean weight 60 kg). The cadavers were prepared for posterior approach THA by an expert consultant orthopaedic surgeon (JC). Bolsters were used to support the cadavers in a lateral decubitus position appropriate for the posterior approach. Prior to surgery, the cadavers were imaged using CT and each acetabulum diameter was measured. An appropriately sized acetabular component was assigned for each sample, corresponding to a diametrical 1 mm interference. The acetabulum was reamed deep enough to expose trabecular bone. Following reaming, a custom surgical mallet (700 g) and introducer were used to impact the acetabulum cup into the reamed cavity. The design of the introducer replicated a typical straight introducer with a clamping mechanism designed to couple rigidly to the acetabular component.

Both the surgical mallet and the introducer were fitted with passive infrared marker arrays. The markers were tracked throughout the impaction procedure using a stereo camera motion tracker (fusionTrack 500; Atracsys, Switzerland). The position and rotation of each marker array were captured at an acquisition rate of 355 Hz, with a root mean square (RMS) error of 90 μm (up to 2 m). A passive marker probe was used to digitize the position of the key features of both the mallet and introducer relative to the global coordinate system. This included the centre of the mallet strike faces and the centre of the handle base. On the introducer, this included the centre of the strike face and the centre of the cup clamp end face. The surgeon (JC) was instructed to impact the acetabulum cup with 24 strikes in total, split into three consecutive groups of strikes of low (eight strikes), medium (eight strikes), and high (eight strikes) effort. The mean velocities of low/medium/high strike effort groups were previously reported and corresponded to 3.9 m/s, 6.1 m/s, and 7.5 m/s, respectively.^16^

### Data analysis

**Fig. 1:**
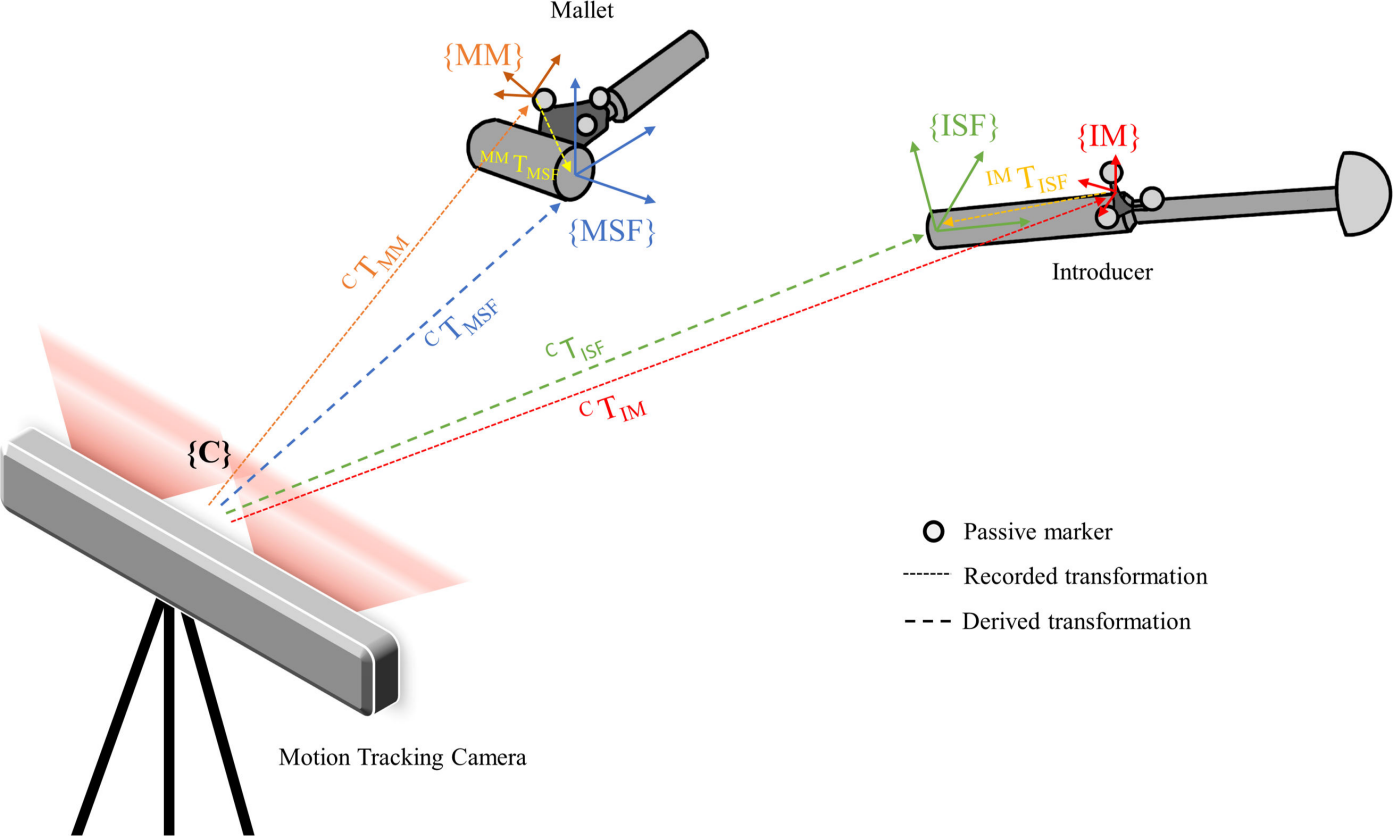
The position and rotation of the mallet strike face (^C^T_MSF_) and the introducer strike face (^C^_T_^ISF^) relative to the camera tracking coordinate system ({C}).

A script was created on MATLAB (2021a; Mathworks, USA) to analyze the positional and rotational data of the mallet and introducers. The motion tracking data were recorded in the form of homogenous transformation matrices, which give the position and rotation of the mallet marker array ($CT_{MM}$) and the introducer marker array ($CT_{IM}$), relative to the global frame of the camera system ({C}). Using the digitized positions of the mallet features, a transformation matrix of the mallet strike face, relative to the mallet marker array, was derived ($MT_{MSF}$). This was repeated with the introducer to derive the introducer strike face relative to the introducer marker array ($IMT_{ISF}$). The position of the mallet strike face relative to the camera coordinate system ($CT_{MSF}$) and the position of the introducer strike face relative to the camera coordinate ($CT_{ISF}$) could then be calculated at each point in time using Equation 1 and Equation 2, respectively (Figure 1).

Equation 1 $$CT_{MSF}=CT_{MM}MMT_{MSF}$$

Equation 2 $$CT_{ISF}=CT_{IM}IMT_{ISF}$$

### Mallet velocity and strike event identification

To remove random tracking error, a third Order Butterworth filter (8,000 Hz cut-off) was applied to the displacement data,^16^ and the velocity was derived from the numerical differentiation of the mallet strike face position with regard to time. Strike events were identified as peak velocity events above a threshold velocity (2.0 ms^-1^). Strike events were manually adjusted when peak velocity occurred before the actual strike event.

### Strike parameters

Three main parameters of the strike event were measured: 1) strike vector angle – the angle of the mallet strike vector, at the point of impact, relative to the central axis of the introducer; 2) strike offset – the distance between the centre of the introducer axis and the centre of the mallet axis at the point of impact; and 3) mallet face angle – the angle of the mallet head strike face relative to the introducer strike face.

**Fig. 2:**
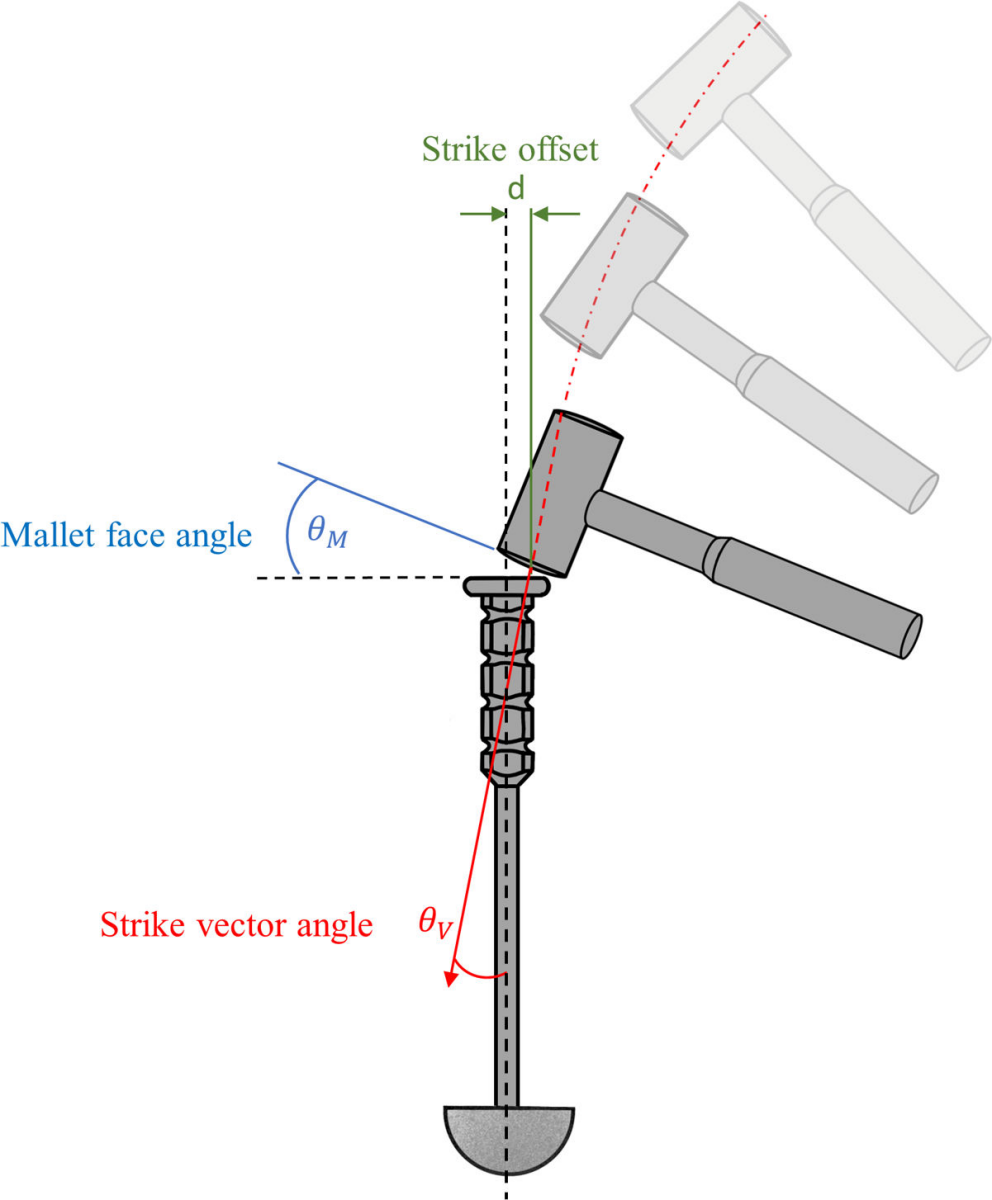
Three strike parameters were measured in post-study analysis of the motion capture data. Strike vector angle, strike offset, and mallet face angle.

These parameters are shown in Figure 2. The percentage of energy wasted, due to the strike vector inaccuracy, was calculated according to Equation 3 and previously reported strike energies.^16^ Wasted energy refers to the portion of energy generated by the strike vector when it deviates from a collinear direction with the introducer, indicating energy not contributing to the advancement of the implant into the joint.

Equation 3 $$Percentage\;wasted\;strike\;energy\;\left(J\right)=\;\;\frac{strike\;energy-\;\left(strike\;energy\;\times cos\left(strike\;angle\right)\right)}{strike\;energy}$$

### Statistical analysis

One-way analysis of variance (ANOVA) testing was carried out on the three independent variable groups: low-, medium-, and high-strike effort. Where significance was found, a post-hoc investigation was performed using Bonferroni correction. Statistical analysis was performed using Prism (v9.0, GraphPad Software, USA).

## Results

### Strike vector angle

**Fig. 3:**
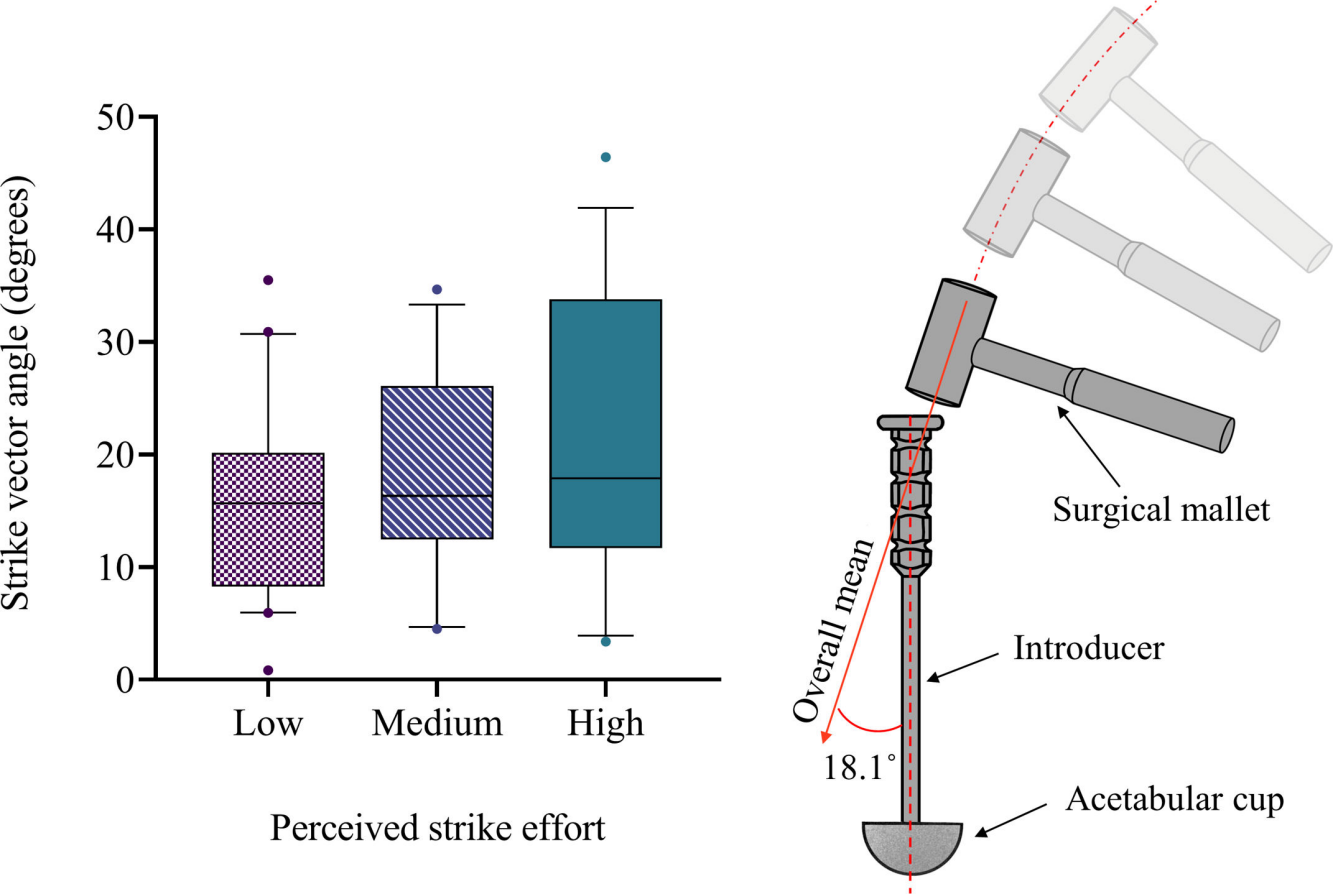
Strike vector angles for low, medium, and high strike efforts during cadaveric total hip arthroplasties. There was no change in strike vector with strike effort, however the mean angle was large. The box represents the interquartile range, the centre line represents the median, and the whiskers represent the 5th to 95th percentile range.

**Fig. 4:**
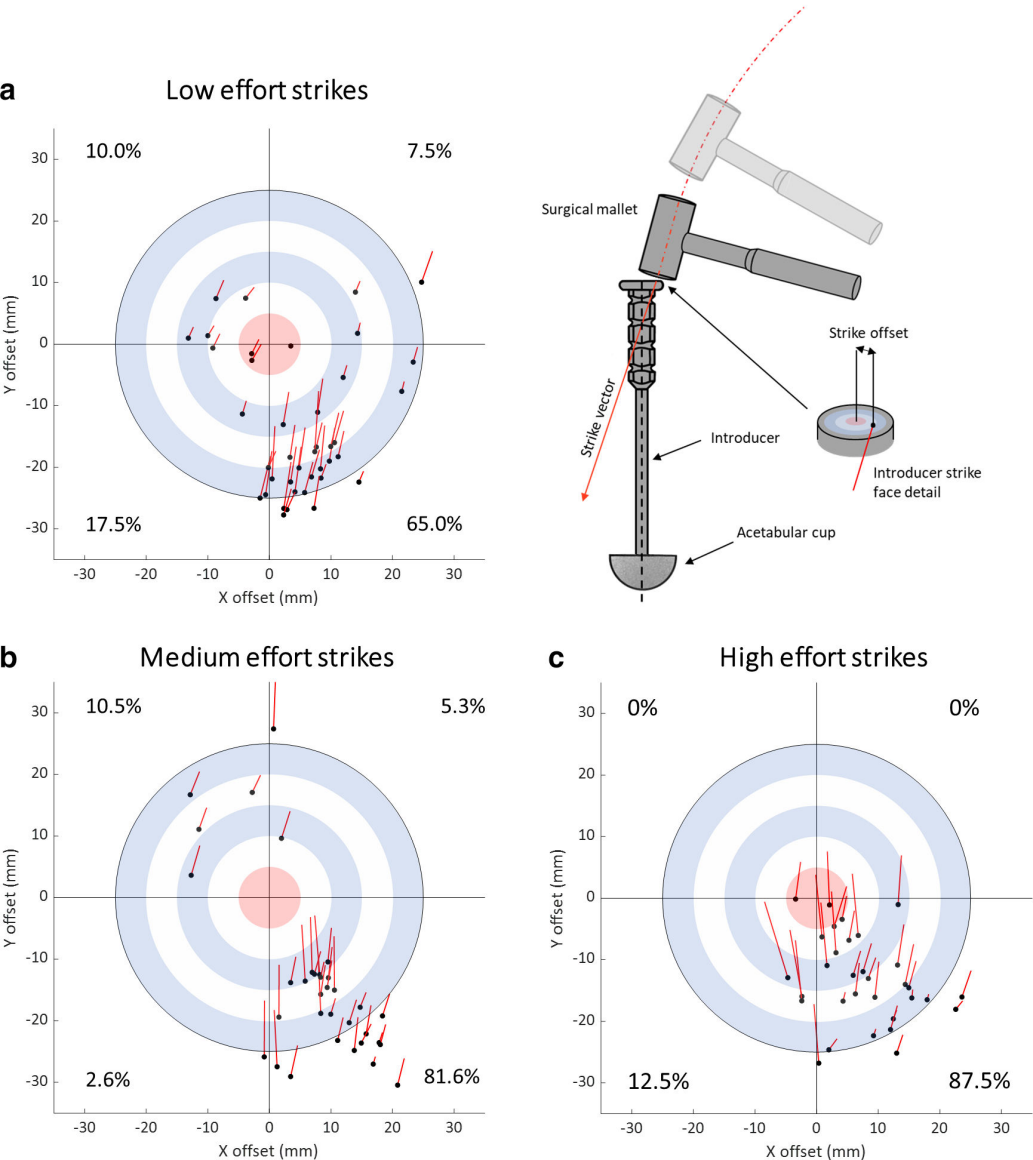
Strike offset and strike vector direction shown, with each strike presented on a target representing the strike face of the introducer (from above). The centre of the target is aligned with the central axis of the introducer, and each point represents the centre of the mallet face during each impact. Each line represents the direction and magnitude of the strike vector off-axis component. a) Mallet impacts for the low-effort impactions. b) Mallet impacts for the medium-effort impactions. c) Mallet impacts for the high-effort impactions.

The vector of the mallet strike was misaligned by a mean angle of 18.1° relative to the axis of the introducer (Table I), and this angle was not affected by the strike efforts (i.e. low, medium, high efforts) (F(2,107) = 2.654, p = 0.075) (Figure 3). The strike vector angle ranged from 0.9° to 46.4° (overall SD 9.8°). The strike vector directions for low-, medium-, and high-effort groups are shown in Figure 4. There was a dominant trend for the direction of strike vectors to be angled towards the upper quadrants of the data plots. All vectors tended to be within the bounds of a similar plane matching the arc of the swing.

**Table I.** Mean and standard deviation of measured impaction dynamics at varying levels of surgeon-perceived strike efforts during cementless acetabular component insertion.

Strike effort	Strike offset, mm		Strike vector misalignment, °		Mallet face misalignment, °		Wasted strike energy, %		N
	Mean	SD	Mean	SD	Mean	SD	Mean	SD	
Low	18.6	7.0	15.6	8.1	13.8	7.0	4.1	4.4	40
Medium	24.0	5.5	18.4	8.9	15.7	10.4	6.2	6.2	38
High	16.6	7.8	20.9	12.2	16.6	3.5	8.6	8.3	32
Overall	19.9	8.7	18.1	9.8	15.2	7.7	6.3	6.1	110

SD, standard deviation.

### Strike offset

The mean strike offset was 19.9 mm (Table I). Strike offset was dependent upon strike effort (F(2,107) = 7.723, p < 0.001). The mean strike offset for medium strike effort (18.6 mm) was 1.3 times greater than low strike effort (24.0 mm), while the mean strike offset for medium strikes was 1.4 times greater than high strike efforts (16.6 mm). There was no difference between the means of the low- and high-effort 154 groups. The variability of strike offsets was high for all strike efforts (overall SD 8.7). Strikes were 155 concentrated in the lower right quadrant of the introducer face with the percentage concentration in that region increasing with strike velocity (65.0% to 87.5%). The strike offsets for the low-, medium-, and high-effort strike groups can be seen in Figure 4.

### Mallet face alignment

**Fig. 5:**
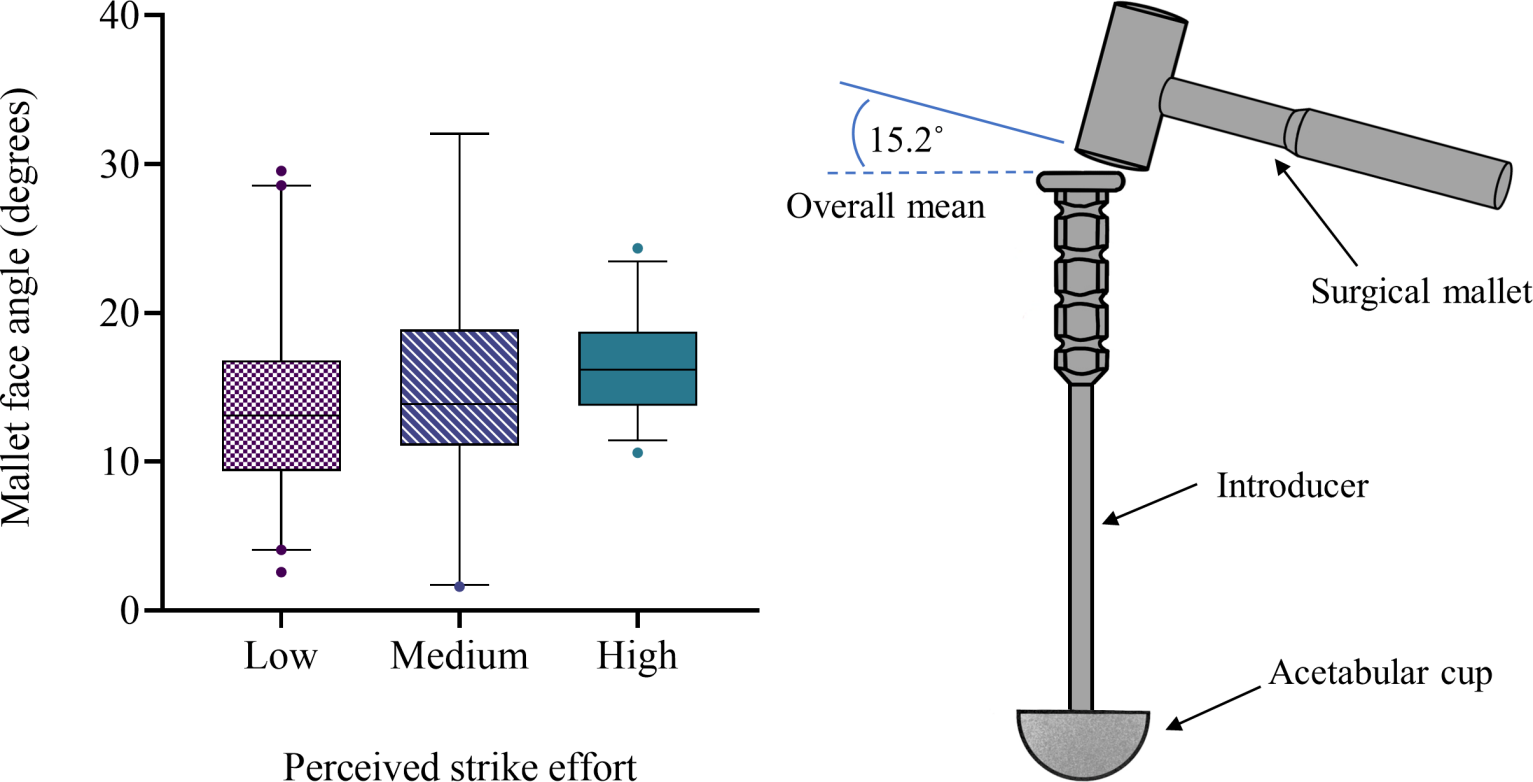
Mallet face alignment angles for low, medium, and high strike efforts during cadaveric total hip arthroplasties. The box represents the interquartile range, the centre line represents the median, and the whiskers represent the 5th to 95th percentile range.

At the moment of impact, the mallet face was misaligned by a mean angle of 15.2° (SD 7.7°) relative to the face of the introducer (Table I). The variability of strike offsets was high for all strike efforts groups. There was no difference in mallet face alignment between the different strike efforts (F(2,104) = 1.260, p = 0.288). These results can be seen in Figure 5.

### Percentage wasted energy

**Fig. 6:**
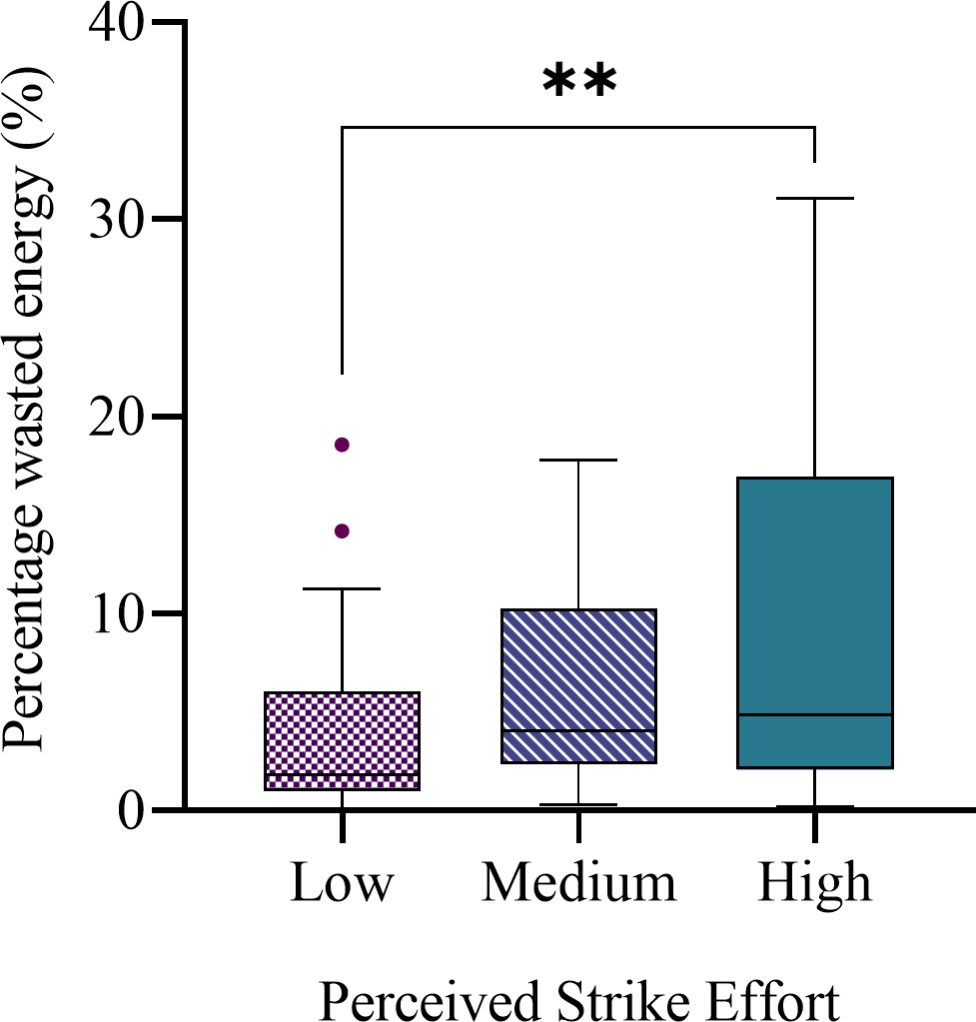
Percentage wasted energy for low, medium, and high strike efforts during cadaveric total hip arthroplasties. The box represents the interquartile range, the centre line represents the median, and the whiskers represent the 5th to 95th percentile range. Significant differences between group means are indicated above the bars.

A mean of 6.3% of the strike energy was wasted during each strike as a result of the strike vector angle misalignment (Table I). There was a difference in the mean percentage wasted energy between different strike efforts (F(2,107) = 4.890, p = 0.009). The mean percentage wasted energy for the high-effort strikes (8.6%) was 2.1 times greater than for the low-effort strikes (4.1%) (p = 0.007). There was no difference in the mean percentage wasted energy between the low- and medium-effort groups and between the medium- and high-effort groups. The wasted energy ranged from 0.0% to 31.7% (SD 6.3%). These results can be seen in Figure 6.

## Discussion

The action of swinging a mallet is a complicated motion recruiting multiple muscle groups in precise coordination. This study has illustrated that a perfectly aligned strike is unusual. The strike vector was misaligned by 15° or more, and the variance for all three strike parameters was high. Thus, the direction of the impact vector in manual impaction lacks both accuracy and precision. The off-axis forces measured in this study can have three undesirable outcomes. First, they will contribute to rotations at the interface between the bone and the acetabular component, damaging the delicate interface and work against achieving primary stability. Second, they could lead to progressive change in the alignment of the acetabular component, as the implant is seated. Third, they waste the surgeon’s energy. Orthopaedic surgery is physically demanding, and wasted energy serves to increase the physicality of surgery, thus contributing to fatigue and risk of injury.^22-25^

From previous studies, it is clear that the force delivered to the impactor varies between surgeons, with a wide range of force values between 3.2 kN and 30.3 kN.^10,11,13,16^ It is important that just the right amount of force and strikes are used to seat a cup to avoid instability, resulting from under impaction, or bone crushing or even fracture, which can result from over-impaction. The window for optimal fixation is narrowed further by the phenomenon of strain deterioration in the cup, compromising the hoop strain in the cup and therefore the fixation.^15^ Excess strikes are shown to increase the risk of cup strain and suboptimal fixation while giving diminishing improvements in push-out force.^14^ In this study, we observe that the strike force’s precision is compromised due to inaccuracy in the strike vector, with up to one-third of the strike energy being wasted.

The precision of the applied force is only one aspect of a high-quality impaction strike. Impaction strikes should result in the fixation of the implant without compromising the orientation of the cup. Hip dislocation is a life-altering complication of THA and a common cause for revision in the USA (17%).^26^ Dislocation can be related to cup orientation.^27^ Suboptimal cup orientation may be due to differences in planning and surgical technique,^28^ and the current study indicates a further consideration may need to be impaction.

In this study, we identified three distinct modes of variability in impaction strikes, each with the potential to induce rotation at the implant-bone interface. First, the strike offset generates a moment composed of the force multiplied by the distance from the introducer axis. Second, the mallet face angle generates a component of force perpendicular to the axis of the introducer. What happens to this component of force depends on the shape of the mallet face and introducer strike surface. A curved mallet face and flat introducer surface would stop this component of force being transmitted to the introducer, however a flat mallet face and curved introducer surface means that this off-axis force is turned into a moment composed of the force multiplied by the length of the introducer. Third, a non-axial strike vector generates a moment composed of the off-axis component of the force times the length of the introducer. Combined, these three variations in strike dynamics form an intricate dynamic interaction which may have compounding effects on the resulting moment, or they may neutralize each other. In this study, strikes trended towards an offset and strike vector combination that will, theoretically, create a moment in opposing directions. The resulting moment is therefore hard to predict.

The underlying cause of the variability relates to the surgeon’s technique, their dominant hand, and their position relative to the patient, which can be affected by being a left or right hip and the surgical approach used. Notably, the systemic bias observed in our results for the strike offset occurring in the same quadrant may be attributed to the surgeon’s hand dominance and surgical approach. Conversely, strikes occurring in the opposing quadrants may be an attempt by the surgeon to correct any migration of the cup during previous strikes.

Manual impaction is a physical activity and the strike energy may pose a long-term occupational hazard. Upper limb injuries can be an issue for surgeons; an occupational hazard study found that over 32% of questioned orthopaedic surgeons suffered from shoulder overuse disorders.^25^ Reducing the energy delivered by the surgeon during impaction would therefore be beneficial. Impact energy can be as high as 18 J in hip arthroplasty.^16^ At this strike energy, our wasted energy data indicate that up to 5.3 J of this energy (30%) may be wasted by the surgeon during a single strike.

This study has several limitations. The observations were limited to a single surgeon; a multi-surgeon study, with surgeons of different experience levels or different physical strengths, would give a greater understanding of variance across the surgical profession. The observations were also limited to three cadaveric specimens. Live-patient surgery presents a highly challenging environment to integrate the specialist data acquisition equipment required for this study, and presents an unnecessary risk to the patient. The lab-based cadaveric trials provided a controlled environment that maximized data acquisition while maintaining a good representation of the surgical set-up by using full-body cadavers and the same bolstering method used in surgery. A further limitation of this study is that the actual strike event of each mallet blow is not captured due to the acquisition rate of the motion tracking system. This is an inherent limitation of all motion tracking systems, however due to the high momentum, the path of the mallet is predictable and can be extrapolated using mallet locations captured prior to the strike event.

Additionally, a further limitation of this study is the absence of a marker array on the pelvis. The inclusion of such a marker array would have enabled precise evaluation of the cup position throughout the impaction process. These additional data could facilitate a more in-depth analysis of the impact of each strike misalignment on the final cup position. Future studies incorporating pelvic marker arrays could provide valuable insights into the nuanced relationship between impaction technique and implant placement, enhancing our understanding of the surgical dynamics involved.

This study has quantified the accuracy and precision of the force vector in manual impaction. The variability measured could potentially be improved through training provided by the orthopaedic industry as a means to reduce the risks associated with off-axis impaction strikes. An alternative approach to reducing these risks could be the provision of a powered impactor instrument. The advantage of such a tool would be to deliver on-axis impact strikes, thus overcoming the various issues identified in the current study. This tool could also reduce the physicality of the procedure, which may make the orthopaedic speciality attractive to more diverse groups of surgical trainees.

This study identified three modes of variability in impact strikes relative to the axis of the introducer: offset distance, mallet face angle, and strike vector. These three variables have the potential to introduce a moment to the introducer and introduce micro-rotations at the implant-bone interface. These micro-rotations could cause unnecessary damage to the press-fit of the implant and also contribute to unintentional change of cup alignment. These results highlight the need for training to improve the precision and accuracy of manual impaction techniques, and exploration of powered impaction instruments that can maintain a linear impaction direction.

## Data Availability

The data that support the findings of this study are available to other researchers from the corresponding author upon reasonable request.

## References

[1] Ben-Shlomo Y Blom A Clark E et al. The National Joint Registry 19th Annual Report 2022 London National Joint Registry https://reports.njrcentre.org.uk/Portals/0/PDFdownloads/NJR%2019th%20Annual%20Report%202022.pdf 36516281

[2] No authors listed The Eighth Annual Report of the AJRR on Hip and Knee Arthroplasty 2021 American Joint Replacment Registry https://www.aaos.org/registries/publications/ajrr-annual-report

[3] No authors listed Hip, Knee and Shoulder Replacement Lay Summary Annual Report 2021 Adelaide Australian Orthopaedic Association National Joint Replacement Registry 2021 https://aoanjrr.sahmri.com/documents/10180/712282/2021+Hip+Knee+Shoulder+Replacement+Lay+Summary/f649829d-294b-43c0-9b30-d38ff3aff534#:~:text=This%20year’s%20report%20involved%20the,and%20including%2031%20December%202020

[4] Illgen R Rubash HE The optimal fixation of the cementless acetabular component in primary total hip arthroplasty J Am Acad Orthop Surg 2002 10 1 43 56 10.5435/00124635-200201000-00007 11809050

[5] Sundfeldt M Carlsson LV Johansson CB Thomsen P Gretzer C Aseptic loosening, not only a question of wear: a review of different theories Acta Orthop 2006 77 2 177 197 10.1080/17453670610045902 16752278

[6] Spears IR Pfleiderer M Schneider E Hille E Morlock MM The effect of interfacial parameters on cup-bone relative micromotions. A finite element investigation J Biomech 2001 34 1 113 120 10.1016/s0021-9290(00)00112-3 11425070

[7] Goodman SB The effects of micromotion and particulate materials on tissue differentiation. Bone chamber studies in rabbits Acta Orthop Scand Suppl 1994 258 1 43 10.3109/17453679409155227 8042498

[8] Maniatopoulos C Pilliar RM Smith DC Threaded versus porous-surfaced designs for implant stabilization in bone-endodontic implant model J Biomed Mater Res 1986 20 9 1309 1333 10.1002/jbm.820200907 3782184

[9] Duyck J Vandamme K Geris L et al. The influence of micro-motion on the tissue differentiation around immediately loaded cylindrical turned titanium implants Arch Oral Biol 2006 51 1 1 9 10.1016/j.archoralbio.2005.04.003 15922992

[10] Fritsche A Bialek K Mittelmeier W et al. Experimental investigations of the insertion and deformation behavior of press-fit and threaded acetabular cups for total hip replacement J Orthop Sci 2008 13 3 240 247 10.1007/s00776-008-1212-z 18528658

[11] Kroeber M Ries MD Suzuki Y Renowitzky G Ashford F Lotz J Impact biomechanics and pelvic deformation during insertion of press-fit acetabular cups J Arthroplasty 2002 17 3 349 354 10.1054/arth.2002.30412 11938513

[12] Vogel D Rathay A Teufel S et al. Experimental analysis of insertion torques and forces of threaded and press-fit acetabular cups by means of ex vivo and in vivo measurements Acta Bioeng Biomech 2017 19 3 155 163 29205219

[13] West C Fryman JC Cadaveric measurement of impact force on total hip arthroplasty surgical instrumentation Am Soc Biomech 2008, Annu Conf Ann Arbor 2008 Michigan, USA

[14] Doyle R van Arkel RJ Jeffers JRT Effect of impaction energy on dynamic bone strains, fixation strength, and seating of cementless acetabular cups J Orthop Res 2019 37 11 2367 2375 10.1002/jor.24418 31317554 PMC6851739

[15] Doyle R van Arkel RJ Muirhead-Allwood S Jeffers JRT Impaction technique influences implant stability in low-density bone model Bone Joint Res 2020 9 7 386 393 10.1302/2046-3758.97.BJR-2019-0303.R1 32793333 PMC7393184

[16] Doyle R Boughton O Plant D Desoutter G Cobb JP Jeffers JRT An in vitro model of impaction during hip arthroplasty J Biomech 2019 82 220 227 10.1016/j.jbiomech.2018.10.030 30420174

[17] Crosnier EA Keogh PS Miles AW A novel method to assess primary stability of press-fit acetabular cups Proc Inst Mech Eng H 2014 228 11 1126 1134 10.1177/0954411914557714 25384445

[18] Cummins F Reilly PO Flannery O Kelly D Kenny P Defining the impaction frequency and threshold force required for femoral impaction grafting in revision hip arthroplasty. A human cadaveric mechanical study Acta Orthop 2011 82 4 433 437 10.3109/17453674.2011.594228 21689068 PMC3237033

[19] Ruhr M Baetz J Pueschel K Morlock MM Influence of acetabular cup thickness on seating and primary stability in total hip arthroplasty J Orthop Res 2022 40 9 2139 2146 10.1002/jor.25232 34855229

[20] Ruhr M Huber G Niki Y Lohner L Ondruschka B Morlock MM Impaction procedure influences primary stability of acetabular press-fit components Bone Joint J 2023 105-B 3 261 268 10.1302/0301-620X.105B3.BJJ-2022-1011.R1 36854327

[21] Bishop NE Höhn JC Rothstock S Damm NB Morlock MM The influence of bone damage on press-fit mechanics J Biomech 2014 47 6 1472 1478 10.1016/j.jbiomech.2014.01.029 24503049

[22] Abbruzzese K Valentino AL Scholl L et al. Physical and mental demand during total hip arthroplasty Orthop Clin North Am 2022 53 4 413 419 10.1016/j.ocl.2022.06.005 36208884

[23] Peskun C Walmsley D Waddell J Schemitsch E Effect of surgeon fatigue on hip and knee arthroplasty Can J Surg 2012 55 2 81 86 10.1503/cjs.032910 22269219 PMC3310761

[24] Davis WT Sathiyakumar V Jahangir AA Obremskey WT Sethi MK Occupational injury among orthopaedic surgeons J Bone Joint Surg Am 2013 95-A 15 e107 1 10.2106/JBJS.L.01427 23925752

[25] Vajapey SP Li M Glassman AH Occupational hazards of orthopaedic surgery and adult reconstruction: A cross-sectional study J Orthop 2021 25 23 30 10.1016/j.jor.2021.03.026 33897136 PMC8058611

[26] Gwam CU Mistry JB Mohamed NS et al. Current epidemiology of revision total hip arthroplasty in the United States: national inpatient sample 2009 to 2013 J Arthroplasty 2017 32 7 2088 2092 10.1016/j.arth.2017.02.046 28336249

[27] Lewinnek GE Lewis JL Tarr R Compere CL Zimmerman JR Dislocations after total hip-replacement arthroplasties J Bone Joint Surg Am 1978 60-A 2 217 220 10.2106/00004623-197860020-00014 641088

[28] Moskal JT Capps SG Scanelli JA Improving the accuracy of acetabular component orientation: avoiding malpositioning: AAOS exhibit selection J Bone Joint Surg Am 2013 95-A 11 e761 10 10.2106/JBJS.L.00685 23780546

